# Editorial

**Published:** 1844-03-09

**Authors:** 


THE MEDICAL EXAMINER.

PHILADELPHIA, MARCH 9, 1844.

### BOND'S PLACENTAL FORCEPS.

At the last meeting of the Philadelphia College of Physicians—after giving some account of the mechanical means heretofore proposed, or now in use, for the removal of the placenta, in those cases of abortion where it is retained and attended with obstinate hæmorrhage—Dr. Bond laid before the College a *placental forceps*, which he had recently contrived.

The instrument is about ten inches long, curved laterally on a radius of about twelve inches. The blades are about an inch and a half longer than the handles, and terminate in an oval expansion half an inch wide. The handles and blades, including the edges of the oval expansion, are rounded or beveled off, very much like those of his œsophagus forceps, so as to preclude all probability of wounding or pinching any of the soft parts. The inner part of the oval expansion is made hollow and rough, so as to maintain a secure grip upon the body embraced. These precise dimensions, as to length and curvature, are not essential, but will probably be found as convenient as any.

The Professors of Obstetrics in the University of Pennsylvania and in Jefferson Medical College have exhibited the instrument to their respective classes, with very decided commendations.

---

### MEDICAL SCHOOLS.

The following pleasant remarks on the multiplication of Medical Colleges in the Valley of the Mississippi are from the last number of the *Western Journal of Medicine and Surgery.* As they are not very inapplicable to the "Atlantic Strip," also, we have concluded to transfer them to the Examiner, for the *amusement*, and perchance instruction, of its readers. We have always been the advocate of perfect freedom of competition in the trades and occupations of human society, and therefore make no complaints against those who act upon that principle in the business of giving medical instruction. But to *give instruction* and to *confer degrees*, are very different things, and it is time that the distinction was broadly made. There is no *necessary* connexion between them, and the public interest and the honor of the profession would be greatly promoted on their separation. As matters now are, there is too much reason to believe that the *diploma*, instead of being awarded as the premium of merit, is too frequently proffered as the price of patronage on the one part, and sought for as the veil for ignorance and imposture on the other; hence it is, that so much hot haste is seen in getting up medical Colleges all over the country; not by the communities for whose benefit they are presumed to be established, but at the instance and for the particular purposes of individuals whose pretensions have been overlooked, or possibly not discovered, by existing institutions. Such, however, appears to be the spirit of the age in which we live, and the genius of our political institutions, that little is to be hoped for in the way of governmental restraint. Whether the moral feeling of the profession can accomplish any reform in this respect, is more than doubtful, under the circumstances in which we are placed.

The Mississippi Valley is remarkable for many things, but will soon be as much so for its medical colleges as for aught else. These multiply astonishingly. They grow as rapidly as Jonah's gourd. One lies down at night to rest from a hard day's toil, and after a deep sleep, such as fell upon Adam, he wakes, and lo! by his side is a medical college, "complete from turret to foundation-stone." They come not singly merely, but by twos and threes—in populous cities, and amid "populous solitudes of bees and birds" as well. Not quicker or more sudden did Roderic Dhu's archers, at blast of bugle-horn, start up from rock and tree, than do these institutions appear at trumpet or even penny-whistle call of some aspiring grandson of Apollo. With one flourish of the serpent-bound staff of him who worshipped

> In Argolis, beside the echoing sea, they come, thick-clustering over the land,

> As when the potent rod
> Of Amram's son, in Egypt's evil day,
> Wav'd round the coast, up-call'd a pitchy cloud
> Of locusts.

We have now a complete chain of them, enough to make a veritable *cordon sanitaire.* Disease is hemmed in—is "cabined, cribbed, confined"—and can have neither egress nor ingress without challenge from trusty sentinel. Tracing them on the map, they make a figure resembling an ill-shaped battledore. Beginning with that at New Orleans, where, as stated in another paragraph, a new college building is just completed, we go to St. Louis and find two more; next to Jacksonville, where there is a fourth; then to Chicago, and count a fifth; turning thence down the lake shore, we find a sixth at Laporte; proceeding to the southern shore of Lake Erie, Cleveland gives us a seventh, and Willoughby, eighteen miles distant, an eighth; crossing the country and following the Ohio, Cincinnati gives the ninth; a *detour* to Lexington, and we have a tenth; Louisville makes the eleventh; another *detour* to Nashville completes the dozen; and if we count one that is in a formative stage at Madison, Indiana, we have a clean baker's dozen. Seven States containing 13 medical colleges, some of which are almost within call of each other!

---

### MEDICAL INSTITUTE OF LOUISVILLE, (Ky.)

The Western Journal announces that "Dr. Cook, the venerable Professor of Theory and Practice," has signified to the board of managers his determination to retire from the School at the close of the present session; and that the board contemplate reducing the number of Chairs to seven. We learn, from other sources, that Prof. Drake is to be transferred to the Chair now occupied by Dr. Cook, and that the one now held by him is to be abolished.

---

The New York Journal of Medicine, boasting of the resources of that city, says,—"Its anatomical opportunities are *so vast* that it furnishes supplies to the Medical Schools from Maine to New Orleans." Whether this *vast* supply of the "*materiel*" is owing to the insalubrity of the place, or the want of skilful physicians, is not stated.

